# Editorial: Forest Genomics and Biotechnology

**DOI:** 10.3389/fpls.2019.01187

**Published:** 2019-10-11

**Authors:** Isabel Allona, Matias Kirst, Wout Boerjan, Steven Strauss, Ronald Sederoff

**Affiliations:** ^1^Centro de Biotecnología y Genómica de Plantas (CBGP, UPM-INIA), Universidad Politécnica de Madrid (UPM) - Instituto Nacional de Investigación y Tecnología Agraria y Alimentaria (INIA), Campus de Montegancedo, Pozuelo de Alarcón, Madrid, Spain; ^2^Departamento de Biotecnología-Biología Vegetal, Escuela Técnica Superior de Ingeniería Agronómica, Alimentaria y de Biosistemas, Universidad Politécnica de Madrid (UPM), Madrid, Spain; ^3^School of Forest Resources and Conservation, University of Florida, Gainesville, FL, United States; ^4^Department of Plant Biotechnology and Bioinformatics, Ghent University, Ghent, Belgium; ^5^Center for Plant Systems Biology, VIB, Ghent, Belgium; ^6^Department of Forest Ecosystems and Society, Oregon State University, Corvallis, OR, United States; ^7^Department of Forestry and Environmental Resources, North Carolina State University, Raleigh, NC, United States

**Keywords:** tree, forest plantation, conifers, angiosperms, wood

Forest biotechnology can be said to have begun with the construction of the first transgenic tree. A bacterial gene imparting glyphosate resistance (EPSP synthase) was introduced into a hybrid poplar (Fillatti et al., 1987). This achievement required the development of three technologies: gene discovery, gene transfer, and *in vitro* plant regeneration. These powerful tools advanced the investigation of the unique biology of forest trees, including their unusual reproductive features, woody and perennial growth habit, and their mechanisms of adaptation to abiotic and biotic stress. In addition, biotechnology has advanced the practical application of molecular genetics for tree breeding, by expanding options for the production of pulp, wood, and energy products (Huang et al., 1993; Baucher et al., 2010; Allwright and Taylor, 2016).

While biotechnology established the tools necessary to modify genes, genomics provided a new platform for the high-throughput genetic analysis of forest trees. Genomics was founded on two technologies: genetic mapping and DNA sequencing. Genetic mapping provided the location of genes, allowing the association of their position to function. Previously, genetic maps were made using isozyme loci as markers (Conkle, 1980) but the number of loci that could be sampled was limited. Restriction fragment length polymorphisms (RFLP), PCR amplification and high throughput DNA sequencing led to an extraordinary expansion in the regions of the genome that could be surveyed (Botstein et al., 1980; Williams et al., 1990; Vos et al., 1995; Wang et al., 1998). Consequently, genetic maps can now be readily constructed for forest trees where genetic analysis of quantitative traits had been previously impossible (Kirst et al., 2004). Through genetic mapping and segregation analysis, a trait could be readily shown to be monogenic, oligogenic, or polygenic in genetic architecture.

Large scale genome mapping of forest trees was first carried out using haploid genetic analysis using conifer megagametophytes and PCR-based anonymous markers (Carlson et al., 1991; Grattapaglia et al., 1992; O’Malley et al., 1996). The concept of mapping with anonymous dominant or codominant markers was extended to diploid crosses using “pseudotestcross” strategies (Grattapaglia and Sederoff, 1994). As sequencing technology advanced, anonymous markers were replaced by sequence-based markers associated with genes, single nucleotide polymorphisms (SNPs) (Eckert et al., 2009) or variation in repeated sequences (Echt et al., 2011).

Forest biotechnology has been strongly influenced by the human genome project (HGP) (Venter et al., 2001). The new technology of the HGP advanced the studies of many species and was important for species such as forest trees, which had been previously difficult due to large sizes and long generation times. Genome sequencing of forest trees (Tuskan et al., 2006) has brought about a host of new technologies (“omics”) where gene expression and function could be studied for single genes or for large gene families and even for all of the expressed genes in a specific tissue or cell type. Omics have been developed in tree species for populations of RNA molecules (transcriptomics), proteins (proteomics), and metabolites (metabolomics). All of these methods have been or are being applied to forest tree species (Wagner et al., 2012; Wang et al., 2014; Wang et al., 2018).

While the new “omics” technologies characterize the activity of biological systems, the understanding about the relationship among them is complex and require mathematical modeling to provide predictive power (Wang et al., 2014). Systems and synthetic biology of forest trees reflect the growing collaboration of engineering and molecular biology. Systems biology integrates levels of information and technology deriving from genomics, such as genome sequencing, transcriptomics, epigenomics, proteomics, metabolomics, and imaging. While systems biology uses information from existing species, synthetic biology goes beyond what already exists in nature by reassembling or inventing novel gene sequences and functions. The goals of systems and synthetic biology are to improve the efficiency of metabolic flux, to redesign pathways, or to create novel ones.

A thorough understanding of metabolic and developmental pathways may not only provide innovations in systems design, but could ultimately prove critical for survival of natural and planted forests. Forests around the world continue to be threatened by an increase in the introduction of nonnative pests and pathogens due to world trade and travel. Epidemics of native species of pests and pathogens have also increased due to the destabilizing effects of climate change, which imposes increased abiotic stress on tree populations. Many species of forest trees may soon be lost, affecting entire ecosystems and leading to loss in ecosystem services and biodiversity (NASEM, 2019). Biotechnology could increase our understanding of host pathogen interactions and could also aid in the development of new genotypes able to resist new biotic and abiotic stresses. This will require major new allocation of resources to studies of forest tree biology and the genetic modification of forest tree populations require major changes in highly restrictive regulations and preclusion from markets (Strauss et al., 2015)

This volume is organized in six sections. (1) Gene Discovery, (2) Gene Transfer and Genetic Engineering, (3) Genetic Mapping and Quantitative Trait Analysis, (4) Growth and Development, (5) Biotic and Abiotic Stress, and (6) Cyberinfrastructure.

(1) Gene Discovery. Great attention has been paid to discovery and manipulation of genes involved in wood formation. Motivated by industrial activity in pulp and paper processing, and by the use of wood for biofuels and solid wood products, it has become critical to identify and characterize genes involved in the chemical and physical properties of wood. Wang et al. (in this volume) have reviewed the status of protein–protein interactions in lignin precursor biosynthesis, and Chanoca et al. (in this volume) have reviewed the efforts made to reduce biomass recalcitrance by engineering lignin quantity and composition. Engineering of noncellulosic polysaccharides in wood is discussed by Donev et al. (in this volume), with the goal of modifying wood for chemical content (particularly sugars) and physical properties, which affect the interactions of hemicelluloses with lignin and cellulose. Many forest trees accumulate high levels of secondary metabolites for defense against pests and pathogens. Conifers and other trees such as eucalypts accumulate terpenes in wood that can be extracted and utilized as a renewable chemical feedstock. Peter (in this volume) has reviewed the status of genetic engineering and breeding approaches to increase the abundance of terpenes and thereby increase the value of plantation forest trees. Myburg et al. (in this volume) integrated systems biology, systems genetics, and synthetic biology to propose a new paradigm for the production of chemical feedstocks from woody biomass and for a multitude of other wood products.

(2) Gene Transfer and Genetic Engineering. The slow and inefficient transfer, incorporation, and expression of specific genes into forest trees continues to be a major barrier to progress. Even more demanding is the subsequent requirement for the transformed cell to dedifferentiate, divide, and regenerate organs or embryos expressing the inserted gene. Only a small number of tree genotypes and species have *in vitro* regeneration systems able to support the stress of DNA insertion and express the plasticity needed for embryogenesis or organogenesis. Nagle et al. (in this volume) have reviewed the challenges and opportunities existing for DNA transformation in forest trees. Use of development-stimulating genes such as WUSCHEL appear highly promising, as do *in vivo* approaches. Bewg et al. (in this volume) summarized the status of gene editing in trees using CRISPR-cas9 technology. CRISPR technology is highly efficient for making knock-outs and other alterations, with few off-target effects in trees. Sterility has been the strategy to mitigate gene flow from future plantations that contain genetically modified trees. The minireview by Fritsche et al. (in this volume) outline approaches to the containment of modified genes, and of exotic and invasive forest tree species, by sterility.

(3) Genetic Mapping and Quantitative Trait Analysis. One major benefit of genomics has been the resulting integration of quantitative and molecular genetics. Now that genomic sequencing can, in principle, identify all the genes, the genetic basis of complex quantitative traits can be potentially identified and characterized more directly. Wood properties are under moderate to strong genetic control (Porth et al., 2012) but are also influenced by environmental factors such as season, rainfall, and variation of the gravity vector (Plomion et al., 2001). Du et al. (in this volume) use wood formation as a model system for investigation of the genetic architecture and regulatory mechanisms of quantitative traits in forest trees. They reviewed recent progress in genome-wide association studies (GWAS) of wood properties as a tool for functional genomics and the potential for molecular breeding. Grattapaglia et al. (in this volume) reviewed the application of genomics to tree breeding and describe how genomic information may be used to improve selection. Genomic selection (GS) may allow predictive markers to accelerate the selection of elite genotypes and discovery of the genetic factors contributing to quantitative traits.

(4) Growth and Development. The growth habit and architecture of forest trees has a major effect on the ecological roles and commercial value of forest trees (Zobel and Jett, 1995; Holliday et al., 2017). Busov (in this volume) has reviewed the regulation of crown architecture, secondary growth, wood formation, and adventitious rooting, all complex traits, based on a number of molecular mechanisms.

Chromatin modification is thought to be the basis for a wide spectrum of changes in gene expression in response to developmental or environmental signaling. Only recently have the extent and roles of chromatin remodeling in forest trees been explored. In this volume, Conde et al. have reviewed this progress focusing on the signatures of chromatin regulation during active growth and seasonal dormancy.

Molecular switches, sensitive to day length and temperature, drive poplar phenology. Short days and low temperatures trigger the sequential induction of ethylene and abscisic acid signaling pathways, bud maturation and the establishment of dormancy. Transcriptional profiling and genetic association studies in poplar by Maurya et al. (in this volume) describe a platform for studying how the environment affects the molecular switches that drive phenology.

A major distinction in forest trees is based on the independent origin of gymnosperms (softwoods) and woody angiosperms (hardwoods). Tuskan et al. (in this volume) have described how, as genome sequence information increases and gene function is better understood, much will be learned about the evolution of these very different lineages with respect to their adaptation to variable environments.

(5) Biotic and Abiotic Stress. The world’s forest tree species are under threat from the globalization of pests and pathogens. At the same time, tree species are increasingly susceptible to these threats due to the increased stress from global climate change. Increased efforts are being made to find or create genetic resistance, either through breeding or genetic engineering. Naidoo et al. (in this volume) have proposed a strategy for the investigation of defense mechanisms that could lead to the development of superior genotypes with enhanced resistance to biotic stress, integrating quantitative and qualitative resistance with the additional contributions of microbial endophytes and the root-associated microbiomes.

Cold hardiness can affect the natural range of forest trees or the establishment of exotic species in new environments (Hinchee et al., 2011). Wisniewski et al. (in this volume) have reviewed the current knowledge of cold hardiness, and the efforts to improve it through transgenic approaches. Cold hardiness is a complex trait involving avoidance, tolerance, seasonal stages, and dormancy.

Nitrogen availability, as ammonia or nitrate, and nitrogen use efficiency are limiting factors for growth and development of forest trees. Nitrogen reserves also affect dormancy and nitrogen cycling in their ecosystem. Nitrogen affects both primary and secondary metabolism. Canovas et al. (in this volume) summarize advances in forest trees for the functional characterization of genes affecting molecular regulation of acquisition, assimilation, and internal recycling of nitrogen.

One of the major effects of climate change is manifested in the water cycles where droughts are expected to be more frequent and more extreme. Polle et al. (in this volume) review approaches to identify genes that could modify drought tolerance through knowledge of the molecular physiology of the responses to drought stress.

(6) Cyberinfrastructure. Databases have become essential to forest biotechnology, as genomic analysis, transcriptomics, metabolomics, and image analysis become accessible tools for genetic engineering and systems biology of forest trees. Wegrzyn et al. (in this volume) describe the existing individual databases, each focus of interest and how they interact to provide synergistic cyberinfrastructure for the forest tree biotechnology community.

Forest genomics and biotechnology is a highly diverse international endeavor, which is advancing rapidly as new technology enables novel approaches and insights. It is an exciting time, and the reviews in this volume provide an excellent update about where things are, and where they are likely to go. Enjoy reading this special issue on Forest Genomics and Biotechnology!

## Author Contributions

All authors participated either in the writing or editing of the editorial.

## Funding

The work in I.As laboratory is supported by Grant PGC2018-093922-B-I00 and SEV-2016-0672 (2017-2021) to the CBGP “Severo Ochoa Programme for Centres of Excellence in R&D” from the Agencia Estatal de Investigación of Spain. WB is indebted to the IWT-FISCH-SBO project ARBOREF (grant number 140894) and the IWT-SBO project BIOLEUM (grant number 130039).

## Conflict of Interest

The authors declare that the research was conducted in the absence of any commercial or financial relationships that could be construed as a potential conflict of interest.
